# Topical glucocorticoid application causing iatrogenic Cushing’s syndrome followed by secondary adrenal insufficiency in infants: two case reports

**DOI:** 10.1186/s13256-022-03659-2

**Published:** 2022-12-08

**Authors:** Nicola Matejek, Johannes Hoos, Paul Martin Holterhus, Markus Bettendorf, Daniela Choukair

**Affiliations:** 1 Paediatric Endocrinology and Diabetes, Children’s Department Klinikum Worms, Worms, Germany; 2 Division of Paediatric Endocrinology and Diabetes, Department of Paediatrics, University Children’s Hospital Heidelberg, Im Neuenheimer Feld 430, 69120 Heidelberg, Germany; 3 Division of Paediatric Endocrinology and Diabetes, Department of Pediatrics I, University Hospital of Schleswig Holstein, UKSH, Campus Kiel, Kiel, Germany

**Keywords:** Topical glucocorticoid application, Iatrogenic Cushing’s disease, Dexamethasone eye drops, Topical application of clobetasol

## Abstract

**Background:**

Iatrogenic Cushing’s syndrome induced by oral and parenteral glucocorticoid administration is a well-known complication. Immediate withdrawal from exogenous steroids can lead to life-threatening adrenal insufficiency. However, Cushing’s syndrome caused by topical treatment with glucocorticoids, such as dexamethasone eye drops or dermal application, is rarely recognized. Young infants in particular are at high risk of suffering from iatrogenic Cushing’s syndrome when treated with highly potent topical glucocorticoids.

**Case presentation:**

We present a 6-month-old Syrian boy with cushingoid face after dermal clobetasol cream treatment and a 2-year-old Iranian girl with severe growth retardation after application of dexamethasone eye drops. Both families have a migration background and language barriers. In both cases no endogenous cortisol secretion was initially detected in serum and in 24-hour collected urine. After dose reduction of glucocorticoids, severity of symptoms was reversible and serum cortisol was detectable.

**Discussion and conclusion:**

Young infants are at high risk of developing Cushing’s syndrome from topically applied highly potent glucocorticoids. Precise recommendations of treatment dosage, duration, and frequency must be given to the parents, and if necessary, with the help of an interpreter. Monitoring of height and weight as well as regular pediatric follow-ups should be scheduled. Physicians should be aware of potential adrenal insufficiency following withdrawal from long-term topical glucocorticoid treatment, and hydrocortisone treatment should be considered.

## Background

Chronic exposure to inappropriate high levels of exogenous glucocorticoids results in Cushing's syndrome (CS) [1]. CS may be characterized by growth failure and weight gain. These children present with “moon face,” truncal obesity, “buffalo hump,” skin bruises, arterial hypertension, hyperglycemia, and proximal muscle wasting [2]. CS in the pediatric age group is very rare, and the vast majority of cases are iatrogenic owing to oral or parenteral administered glucocorticoid hormones. An iatrogenic CS is associated with the systemic daily dosage of 10–12 mg/m^2^ hydrocortisone (corresponding to 0.3 mg dexamethasone) or higher for more than 7 days [2, 3]. Immediate withdrawal from exogenous steroids can lead to life-threatening adrenal insufficiency [3]. However, iatrogenic CS in childhood owing to topical glucocorticoid administration may occur and is probably underreported [4, 5]. Dermal application is used in inflammatory skin diseases [6] or inhalation in obstructive lung diseases [7]. Further, topical glucocorticoids are frequently used as rectal application in inflammatory bowel disease [8], as nose spray in obstructive rhinitis [9], or as eye drops in postoperative ocular inflammations [10]. Systemic side effects of topical glucocorticoid depend on the substance used and are subject to individual resorption conditions [11, 12]. Parents must be fully informed about potential side effects. Herein, we present a case of iatrogenic CS in a 2-year-old infant induced by dexamethasone-containing eye drops and a second case of a 6-month-old infant in whom CS was induced by dermal application of clobetasol.

## Case presentation

### Case 1

The male patient was born at term after an uneventful pregnancy. He was the fifth child of a Syrian refugee family. Several weeks prior to first presentation, they stayed in a Greek refugee camp and he suffered from scabies. After treatment with permethrin, itching persisted and topical treatment with clobetasol propionate 0.05% cream was recommended. The dystrophic boy presented at 6 months of age at our hospital with a cushingoid face and scratch marks on the abdomen. At that time clobetasol propionate 0.05% cream had been applied twice daily for a period of at least 4 weeks. Height standard deviation score (SDS) was −0.26, weight SDS was −1.46, and body mass index was −2.69. Unfortunately, no previous anthropometric values were available. Normal serum concentrations for electrolytes, glucose, and plasma renin activity were determined. Morning cortisol serum concentrations [14 nmol/L (normal range 155–552 nmol)] and adrenocorticotropic hormone (ACTH) concentration [1.3 pmol/L (normal range 2.2–11.0 pmol/L)] were low [measured by liquid chromatography tandem mass spectrometry (LC–MS/MS)]. Additionally, in 24-hour collected urine, free cortisone secretion [2.8 nmol per 24 hours (normal range 8.3–69 nmol)] and free cortisol secretion [< 1 nmol per 24 hours (normal range 8.3–69 nmol)] were very low and not detectable, respectively. Iatrogenic Cushing’s syndrome with subsequent adrenal insufficiency was diagnosed. Therefore, we started oral treatment with 9 mg hydrocortisone per m^2^ body surface per day divided in three doses, and educated the mother on dose adaption in cases of stress situations. Owing to language barrier, the education was given with the help of an interpreter. Local treatment with clobetasol propionate 0.05% cream was ceased. Follow-up 3 weeks after dismissal demonstrated persistent adrenal insufficiency with low morning cortisol serum concentration [17 nmol/L (normal range 155–552 nmol/L)] and ACTH serum concentration [< 1.1 pmol/L (normal range 2.2–11.0 nmol/L)] 12 hours after last intake of hydrocortisone. The cushingoid face diminished and continuation of hydrocortisone treatment was recommended. After this visit the family was transferred to a refugee camp elsewhere in Germany and unfortunately the contact and opportunity for further follow-up were lost.

### Case 2

**Fig. 1:**
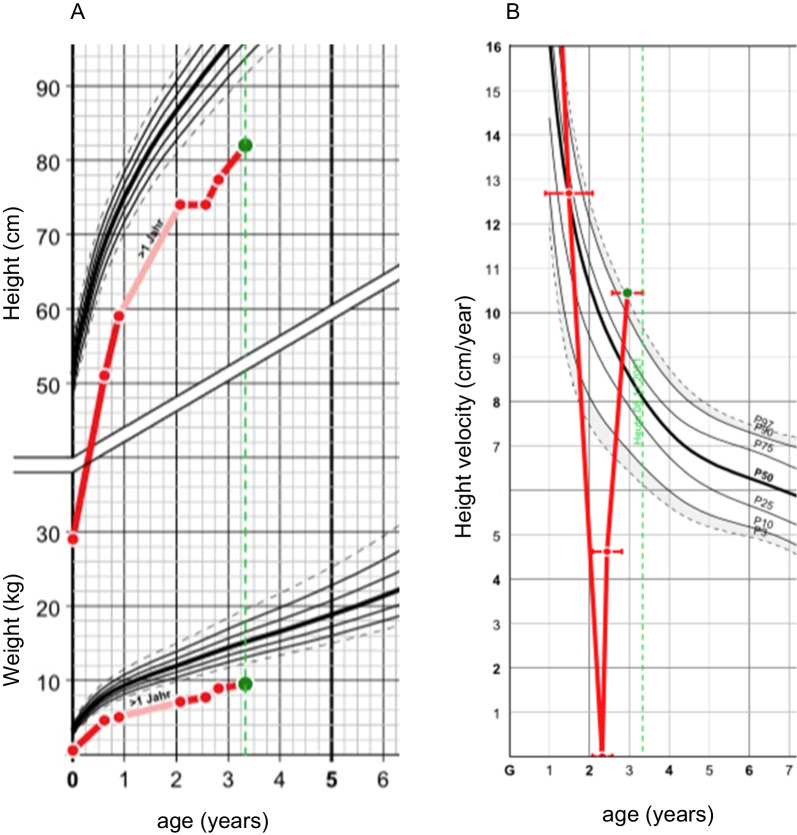
**A** Growth chart of case 2, **B** height velocity of case 2. German references were used for height, weight, and height velocity [27, 28]

A 2-year-old girl was presented for short stature. She was a preterm baby born at 24 + 1 gestational weeks with appropriate weight and length. She was the second daughter of Iranian, unrelated parents. She had many complications in the postnatal period; among others she suffered from a congenital glaucoma and underwent several eye surgeries. The last surgery was in August 2020. After each surgery, she was treated with dexamethasone eye drops for a few weeks in decreasing doses. Up to the first consultation at our clinic in November 2020, she received 4 dexamethasone eye drops (1.3 mg/ml) per day (3 drops left, 1 drop right eye), corresponding to 0.3 mg dexamethasone per day. She presented with short stature (−5.52 SDS) and was dystrophic with a reduced body mass index (14 kg/m^2^, −1.42 SDS). Target height corresponded to the tenth percentile (−1.3 SDS) (Fig. 1a). Pediatric endocrine examination excluded hypothyroidism, growth hormone deficiency, bone mineralization deficiency, malabsorption, nephropathy, hepatopathy, anemia, and chronic inflammation. The bone age was age appropriate. The chromosome analysis and array-based comparative genomic hybridization (array-CGH) were unremarkable. A cerebral magnetic resonance imaging (MRI) showed a hypoplastic corpus callosum, periventricular leukomalacia, and a normal pituitary gland.

Cortisol serum concentrations were undetectable, throughout and low ACTH serum concentrations were measured (Table 1). The adrenal insufficiency was confirmed by ACTH stimulation (cortisol serum concentration at 0 minutes  < 14 nmol/L and after 60 minutes 50 nmol/L) and by corticotropin-releasing hormone stimulation (Table 2). Within 4 weeks we reduced the dosage of dexamethasone eye drops to 0.15 mg/days. The serum concentrations of dexamethasone measured 1.02 nmol/L by LC–MS/MS indicating a clearly relevant resorption of dexamethasone. Four weeks later a normal serum steroid profile was detected by LC–MS/MS indicating regular endogenous glucocorticoid biosynthesis within the adrenals (Table 3).

**Table 1:** Diurnal serum concentrations of cortisol and corresponding ACTH of case 2

Time (hour)	ACTH serum concentrations (2.2–11.0 pmol/L)	Cortisol serum concentrations (155–552 nmol/L)
08:00	6	< 14
11:00	2	< 14
17:00	2.4	< 14
00:00	1.8	< 14 (< 50)

*ACTH*, adrenocorticotropic hormone

**Table 2:** Corticotropin-releasing hormone test of case 2

Time (minutes)	ACTH serum concentrations (2.2–11.0 pmol/L)	Cortisol serum concentrations (155–552 nmol/L)
0	3	41
15	2.2	16.5
30	2.4	36
45	2.9	41
60	–	27.5
90	2	19.3
120	2.4	19.3

*ACTH*, adrenocorticotropic hormone

**Table 3:** Serum steroid profile of case 2 8 weeks after dosage reduction of dexamethasone eye drops

Parameter	Serum concentrations	Reference range
ACTH	3.7	2.2–11.0 pmol/L
Progesterone	0.08	0.1–1.4 nmol/L
17-Desoxycorticosterone	0.49	0.2–1.7 nmol/L
Corticosterone	7.68	0.2–85 nmol/L
Aldosterone	1.07	0.8–2.3 nmol/L
17-Hydroxyprogesterone	0.07	0.7–17 nmol/L
21-Desoxycortisol	0.53	0.1–1.8 nmol/L
11-Desoxycortisol	0.77	0.3–2.8 nmol/L
Cortisol	155.2	155–552 nmol/L
Cortisone	30.9	5–94 nmol/L
Androstendione	< 0.1	0.1–1.9 nmol/L
Testosterone	0.16	0.1–0.65 nmol/L

*ACTH*, adrenocorticotropic hormone

Eight weeks later, the girl presented with catch-up growth (height velocity by 4 SDS) after tapering the dosage of dexamethasone eye drops (0.15 mg/days) and appetite improved (Fig. 1b). The diurnal cortisol secretion determined in saliva was normal (Table 4). Both families have given their written informed consent to publish their case (including publication of images).

**Table 4:** Diurnal cortisol serum concentrations determined in saliva of case 2 8 weeks after dosage reduction of dexamethasone eye drops

Time (hour)	Cortisol saliva concentrations
08:00	1.3 nmol/L
14:00	0.19 nmol/L
22:00	0.17 nmol/L

## Discussion and conclusions

Cushing’s syndrome due to topical administration of glucocorticoids is a rare condition in infants and children, but probably underreported [12]. Exogenous glucocorticoids lead to suppression of hypothalamic–pituitary–adrenal (HPA) axis, and life-threatening addisonian crisis can occur [11]. The first case we presented developed iatrogenic CS after inappropriate and prolonged use of highly potent topical glucocorticoid, that is, clobetasol propionate for treatment of scabies. At least 43 cases with iatrogenic CS from very potent topical steroid usage (clobetasol) in children and adults have been published over the last 35 years. particularly in developing countries [5]. Most patients were infants with diaper dermatitis and were treated for a median duration of 2.75 months (1–17 months) [5]. In all cases, CS was clinically obvious and suppressed cortisol and ACTH levels were detected. After discontinuation of topical steroids, HPA axis recovered after 3.49 ± 2.92 months (1–12 months) [5]. The effect of topical glucocorticosteroids depends on type of corticosteroid and its bioavailability, the vehicle, the integrity of the skin, the use of occlusive dressings, surface area, frequency and duration of treatment, presence of inflammation, and anatomic region [13]. Anatomic regions with a thin epidermis are significantly more permeable to topical steroids than thick-skinned areas [14]. Occlusive dressings will enhance drug resorption, often by a factor up to ten [15, 16]. Our patient received a daily dosage of 2 g/day of 0.05% clobetasol (corresponding to 0.1 g clobetasol per day). The potency of clobetasol is estimated to be 600 times higher compared with hydrocortisone, therefore 1 mg clobetasol corresponds to 600 mg hydrocortisone. It is known that the use of 2 g/day of 0.05% clobetasol propionate can decrease morning cortisol levels after only a few days [17] and use over 100 g/week can lead to the development of features of CS and symptoms of adrenal insufficiency [18]. The patient here (case 1) presumably received this amount. Furthermore, the boy presented with abdominal scratch marks and was malnourished. Presumably these factors and the stressful journey facilitated the development of CS in his case. The reported family has refugee status and only limited Greek, German, or English knowledge, contributing to communication problems. Professional interpreters should be introduced to explain medical details [19].

There are several ophthalmologic indications for topical ocular steroid treatment in children, one being postoperative treatment [10]. Often, an intensive therapy scheme is necessary over several weeks [10]. The systemic resorption of glucocorticoid-containing eye drops depends on the frequency, concentration, and duration of application. In general, the conjunctiva, but also the nasal mucosa via the lacrimal drainage system, is highly resorptive [20]. Therefore, it is advisable to apply finger pressure to the lacrimal sac for 1–2 minutes after instillation of dexamethasone eye drops to decrease the risk of resorption and systemic effects [21]. Our patient initially received 0.3 mg dexamethasone daily. Even after reducing the dexamethasone dosage to 50% of the initial dose, the measured serum dexamethasone concentration was 1.02 nmol/L, indicating systemic resorption. This corresponds to 30-fold potency of hydrocortisone. Interestingly, when dexamethasone eye drops were reduced to 0.15 mg daily, catch-up growth occurred and endogenous cortisol secretion normalized. It is known that a decrease in growth velocity is observed as soon as daily dosages exceed a cortisol equivalent of 10–12 mg/m^2^ body surface/24 hour [22]. Caution is necessary if additional medications are administered. Glucocorticoids are mainly metabolized in the liver via CYP3A4 into inactive compounds and are further eliminated as urinary metabolites. Therefore, comedication of CYP3A4 inhibitors, that is, protease inhibitors, itraconazole, macrolides, and diltiazem can increase the risk of the development of CS from using topical corticosteroids [5]. For evaluation, the adrenal axis ACTH-and CRH-stimulation test, as we have undertaken in case 2, were performed. Ach *et al*. proposed the glucagon stimulation test as a safe alternative test for the assessment of the hypothalamic pituitary adrenal axis [23, 24]. If CS is obvious and iatrogenic adrenal insufficiency is induced, abrupt discontinuation of long-standing glucocorticoid treatment should be avoided [3, 25]. A reduction scheme should be provided and explained in detail to the parents [26]. If a language barrier is obvious, the education should be given with the help of an interpreter. Further compliance should be checked at a follow-up visit to avoid life-threatening complications. The duration of recovery of the HPA axis suppression varies [25]. Therefore, the patient must be educated to increase the hydrocortisone dose as indicated in the personalized emergency pass in case of acute illness or any symptoms resembling addisonian crisis, including vomiting. Patients should immediately go to the hospital to potentially receive parenteral corticosteroids and, if necessary, hemodynamic support.

## Conclusions

Both cases reveal that young infants are at high risk of Cushing’s syndrome from topically applied highly potent glucocorticoids. Therefore, physicians prescribing topical steroids should be aware of such complications and closely follow these patients. A precise application recommendation including dosage, duration, and frequency must be explained to the parents, and if necessary, with the help of an interpreter. Further, physicians should avoid abrupt withdrawal from long-term treatment with glucocorticoids, which can cause adrenal crisis if adrenal insufficiency is present. Instead, careful weaning of topical corticosteroids is indicated as treatment with hydrocortisone should be considered.

## Data Availability

Additional data is available upon request from the corresponding author if in line with the consents.

## Abbreviations

ACTH: Adrenocorticotropic hormone
Array-CGH: Array-based comparative genomic hybridization
CS: Cushing’s syndrome
HPA: Hypothalamic–pituitary–adrenal
LC–MS/MS: Liquid chromatography tandem mass spectrometry
MRI: Magnetic resonance imaging
SDS: Standard deviation score
